# Identification and functional analysis of novel *SOX11* variants in Chinese patients with Coffin-Siris syndrome 9

**DOI:** 10.3389/fgene.2022.940776

**Published:** 2022-07-22

**Authors:** Yu Ding, Jiande Chen, Yijun Tang, Li-Na Chen, Ru-En Yao, Tingting Yu, Yong Yin, Xiumin Wang, Jian Wang, Niu Li

**Affiliations:** ^1^ Department of Endocrinology and Metabolism, Shanghai Children’s Medical Center, Shanghai Jiao Tong University School of Medicine, Shanghai, China; ^2^ Department of Respiratory Medicine, Shanghai Children’s Medical Center, Shanghai Jiaotong University School of Medicine, Shanghai, China; ^3^ Department of Medical Genetics and Molecular Diagnostic Laboratory, Shanghai Children’s Medical Center, Shanghai Jiaotong University School of Medicine, Shanghai, China; ^4^ Shanghai Key Laboratory of Clinical Molecular Diagnostics for Pediatrics, Shanghai, China; ^5^ Shanghai Clinical Research Center for Rare Pediatric Diseases, Shanghai, China

**Keywords:** SOX11, coffin-siris syndrome, missense variants, functional study, phenotypic differences

## Abstract

SOX11 is a transcription factor belonging to the sex determining region Y-related high-mobility group box family that plays a vital role in early embryogenesis and neurogenesis. *De novo* variants in *SOX11* have been initially reported to cause a rare neurodevelopmental disorder, mainly referred to Coffin-siris syndrome 9 (CSS9, OMIM# 615866) which is characterized with growth deficiency, intellectual disability (ID), microcephaly, coarse facies, and hypoplastic nails of the fifth fingers and/or toes. A recent large-scale cohort study suggests that *SOX11* variation would result in a clinically and molecularly distinct disease from CSS. Here, we describe three unrelated Chinese cases with variable phenotype, mainly involving developmental delay, ID, short statute, microcephaly, facial deformities (i.e., prominent forehead, arched eye brow, flat nasal bridge, broad nose and short philtrum), and cryptorchidism. Whole-exome sequencing (WES) revealed three novel heterozygous variants in the *SOX11* gene, including two missense variants of c.337T>C (p.Y113H) and c.425C>G (p.A142G), and one nonsense variant of c.820A>T (p. K142*). Luciferase reporting assay shows that the two missense variants impair the transcriptional activity of the *SOX11* target gene *GDF5*. Additionally, WES uncovered a 4,300 kb deletion involving the region of 1q24.2-q25.1 (hg19,chr1:169,433,149-173,827,682) in patient 1, which also contributes to the condition of the patient. In summary, this is the first report of Chinese cases with *de novo* variants of *SOX11*. Our study partially supports the previous observation that the phenotype caused by *SOX11* variants somewhat differs from classical CSS.

## Introduction

The sex determining region Y (SRY)-related high-mobility group (HMG) box (SOX) family encodes a group of transcription factors that play essential roles in cell fate decisions during many developmental processes (Sarkar and Hochedlinger, 2013). Thus far, a total of eight SOX subgroups (A, B1/B2, C, D, E, F, G, and H) with twenty members have been identified, all of which harbor a highly conserved DNA-binding HMG domains (Bowles et al., 2000). It is well known that dysfunction of the SOX proteins plays a critical role in the occurrence and development of multiple malignant tumors (i.e., hepatocellular carcinoma) *via* transcriptional activation or suppression of distinct downstream targets or signaling pathways (de la Rocha et al., 2014; Grimm et al., 2020; Luo et al., 2022). In addition, germline variants in twelve of SOX members can lead to kinds of human genetic disorders, including *SRY* (OMIM# 480000), *SOX2* (OMIM# 184429), *SOX3* (OMIM# 313430), *SOX4* (OMIM# 184430), *SOX5* (OMIM# 604975), *SOX6* (OMIM# 607257), *SOX8* (OMIM# 605923), *SOX9* (OMIM# 608160), *SOX10* (OMIM# 602229), *SOX11* (OMIM# 600898), *SOX17* (OMIM# 610928), and *SOX18* (OMIM# 601618) (Angelozzi and Lefebvre, 2019).

The *SOX11* gene belongs to subgroup C of SOX family, which locates at 2p25.2 and contains only one exon to encode a small protein comprising 441 amino acids (NP _003099.1; UCSC database, http://genome.ucsc.edu). In 2014, Tsurusaki et al. first demonstrated that *de novo* variants in *SOX11* can result in Coffin-siris syndrome 9 (CSS9, OMIM#615866) in two patients and animal models (Tsurusaki et al., 2014). CSS is usually characterized with growth deficiency, intellectual disability (ID), microcephaly, coarse facies, and hypoplastic nails of the fifth fingers and/or toes (Kosho et al., 2014; Sokpor et al., 2017). In 2016, Hempel et al. confirmed the neural phenotype related to CSS9 in ten patients with *de novo* single nucleotide variants (SNVs) of *SOX11* or microdeletions of the chromosome 2p25.2 containing *SOX11* (Hempel et al., 2016). Along with the identification of new patients, several new clinical features were reported, including coarctation of the aorta (Okamoto et al., 2018), cleft palate (Khan et al., 2018), and glaucoma (Diel et al., 2021). More recently, a large cohort study revealed that developmental delay (DD) or ID, microcephaly, short stature, and low body weight were common characteristics in patients with *SOX11* variants. In addition, ocular malformations (oculomotor apraxia, coloboma, and microphthalmia) and hypogonadotropic hypogonadism were also reported in patients with *SOX11* variation (Al- Jawahiri et al., 2022). These studies indicate phenotypic complexity from *SOX11* variation, and a full understanding of such disease requires the accumulation of additional cases from different ethnic groups.

Here, we reported three unrelated Chinese patients with distinct *de novo* variants in *SOX11* gene which were identified by whole-exome sequencing (WES). Phenotypic analysis showed that most of the clinical features of the three patients were consistent with those reported, but there were still some differences. Additionally, the impact of the two missense variants (Y113H and A142G) on SOX11 protein was investigated by *in vitro* experiments.

## Materials and methods

### Patients

Three unrelated patients (two males and one female) born to physically healthy and non-consanguineous parents were enrolled in this study. Written informed consent has been obtained from each family.

### DNA sequencing

WES was performed in all three patients as previously described (Wang et al., 2014; Wang et al., 2016). Briefly, genomic DNA was extracted from patients’ peripheral blood and was then sheared to create fragments of 150 to 200 bp. Sequencing library was prepared using the SureSelect XT Human All Exon V6 kit (Agilent Technologies, Santa Clara, CA, United States), and sequencing was performed by the Illumina NovaSeq 6000 System (Illumina, San Diego, CA, United States). After base calling, quality assessment, and alignment of the sequence reads to the reference human genome (GRCh37, dbSNP135), all single nucleotide variants (SNVs) and indels were saved as VCF format file which was then uploaded to the QIAGEN Clinical Insight (QCI) Interpret Translational tool (https://apps.qiagenbioinformatics.cn/) for filtering and annotation. The *SOX11* variants identified by WES were validated by Sanger sequencing using the ABI 3700 sequencer (Applied Biosystems, Foster City, CA, United States), in indicated patient and their parents (sequencing primers were available upon request).

### Cell culture

HEK293T cells were grown in Dulbecco’s modified Eagle’s medium supplemented with 10% (v/v) fetal bovine serum (Sigma-Aldrich, St. Louis, MO, United States) and 1% penicillin/streptomycin (Thermo Fisher Scientific, Waltham, MA, United States) in a 5% CO2 incubator at 37°C.

### Plasmids construction and transfection

The wild-type (WT) and mutant *SOX11* cDNA were synthesized by BGI (Shenzhen, China), and were then cloned into pcDNA3.1(+)-N-MYC vector, respectively. The *GDF5* promoter 5′-flanking sequence (−448/+319) was synthesized and cloned into the pGL3-basic vector. Plasmid DNA was transfected into cells using Lipofectamine™ LTX reagent (Thermo Fisher Scientific).

### Immunofluorescence

HEK293T cells were cultured on cover slips in 12-well plate at 80% confluence prior for transfection. 24 h after transfection with the WT or mutant *SOX11* constructs, cells were washed by 1 x PBS and fixed using 4% paraformaldehyde for 15 min at room temperature. Samples were then washed with 1 × PBS three times and blocked in the blocking buffer (1x PBS/5% goat serum/0.3% Triton X-100) for 1 h. Coverslips were incubated with mouse anti-Myc monoclonal antibody (Cell Signaling Technology, Danvers, MA, United States) at 4°C overnight. The cover slips were then mounted on microscope slides using ProLong^®^ Gold Antifade reagent with DAPI (Cell Signaling Technology) and analyzed using a Leica DM6000 fluorescence microscope (Leica Microsystems, Wetzlar, Germany).

### Western blotting

HEK293T cells were seeded into 6-well plate and transfected with the indicated *SOX11* constructs. 24 h after transfection, cells were washed with ice-cold 1x PBS and lysed with SDS lysis buffer (100 mM pH6.8 Tris-HCl, 10% Glycerol, 1% SDS). Whole cell lysates were separated on 10% SDS-PAGE gels, transferred to polyvinylidene difluoride (PVDF) membranes. After blocking in 5% non-fat milk in TBS-T for 1 h, membranes were then incubated overnight at 4°C with mouse anti-Myc monoclonal antibody (Cell Signaling Technology) and mouse anti-beta-Actin monoclonal antibody (Sigma-Aldrich). Proteins were detected using a chemiluminescence system with a horseradish peroxidase conjugated secondary antibody.

### Luciferase reporter assays

*GDF5*-luc plasmid was used to monitor the regulatory role of *SOX11* in modulating the transcription. Briefly, HEK293T cells were seeded into 96-well plate at a density of 3×10^4^ per well. 24 h after culture, the cells were co-transfected with 28 ng *GDF5*-luc, 2 ng pRL-SV40, and 70 ng control or *SOX11* expression vectors. 24 h after transfection, luciferase activity was determined by the Dua-Glo luciferase assay system (Promega Corporation, Madison, WI, United States), and normalized to renilla luciferase.

## Results

### Clinical description of the patients

**FIGURE 1:**
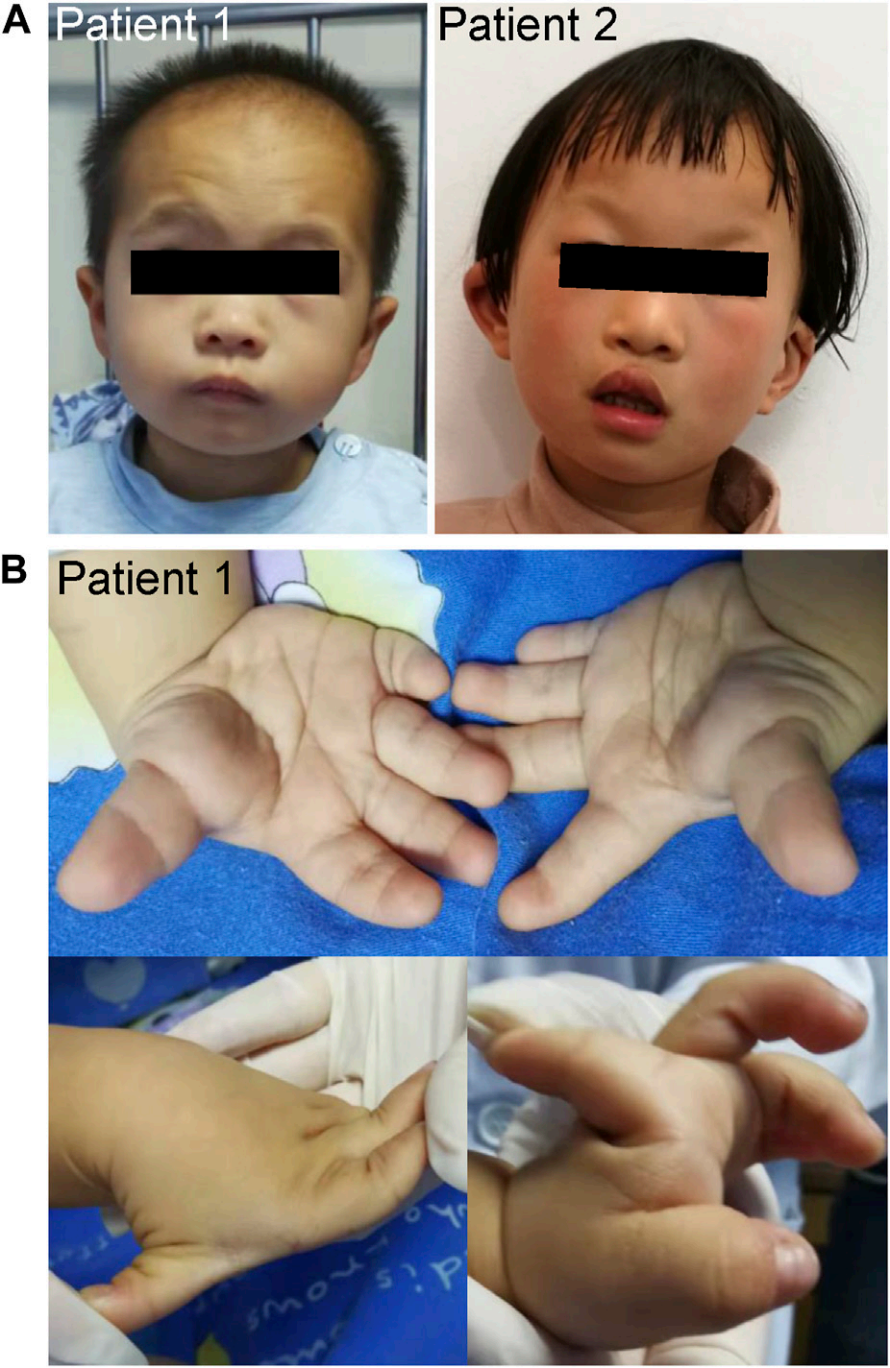
Clinical characteristics of the patients. **(A)** Facial photographs of patients 1 and 2. **(B)** Patient 1 had short fingers, clinodactyly of the fifth finger, and abnormal knuckles activity.

Patient 1 was a male who was born at full-term gestation with a birth weight of 2100 g (−3.1 SD) by spontaneous vaginal delivery. At the stage of pregnancy, intrauterine growth restriction (IUGR) was noticed. At 3-year old, he was referred to our hospital due to growth retardation. Physical examination showed a height of 79 cm (−4.7 SD) and weight of 9.6 kg (−3.3 SD). His hair was sparse and the anterior hairline was high. He presented with facial deformities consisting of prominent forehead, arched eye brows, flat nasal bridge and broad nose, low-set ears, short philtrum, and micrognathia (Figure 1A). He had left cryptorchidism, short fingers, and clinodactyly of the 5th finger. In addition, finger joint laxity was also noticed in the patient (Figure 1B). X-ray showed delay bone age which was 1.5-year old. No DD or ID was found in the patient. During the last follow up, his parents denied the child have mental development problems and refused further assessment, though learning difficulties were mentioned. No special was recorded for the development of the gross motor skills of the patient. The levels of growth hormone, IGF-1, IGF-BP3, LH and FSH were normal. Cranial magnetic resonance imaging (MRI) showed his pituitary gland was thin and oblate. Abdominal ultrasound was normal.

Patient 2 was a 5.5-year old female who was born at full-term gestation with birth weight of 3500 g (0.7 SD) and birth length of 50 cm (0.2 SD) by cesarean section. She was able to sit at 6-month old and walk alone at 20-month old. At the age of 5-year old, she was found with severe speech impairment and moderate ID. Physical examination showed a height of 113 cm (−0.1 SD) and weight of 18.7 kg (−0.3 SD). She had prominent forehead, high hairline, sparse scalp hair, arched eye brows, short philtrum, flat nasal bridge and broad nose, hypoplastic right nasal alar, auricle malformation, everted upper lip, and cleft palate and lip (Figure 1A). X-ray of the bone age, cranial MRI, and the abdominal ultrasound were normal.

Patient 3 was an 8-year old male patient born at full-term gestation by cesarean section. He was admitted to the Endocrinology department due to a slow increase in height, which was less than 4 cm/year for 4 years. His birth weight was 3200 g (−0.3 SD) and birth length was 49 cm (−0.8 SD). Physical examination showed a height of 118 cm (−2.2 SD) and weight of 17 kg (−2.5 SD). He had prominent forehead, high hairline, flat nasal bridge, and bilateral cryptorchidism. Specialist assessment suggested he had DD and mild ID. X-ray showed a two-year delay in his bone age. The levels of growth hormone, IGF-1, IGF-BP3, LH and FSH were normal. Testosterone levels had a good response after HCG stimulation, increasing from less than 0.01–0.28 ng/ml. The cranial MRI and abdominal ultrasound were normal.

We compared the clinical phenotypes of our patients with those previously reported (Tsurusaki et al., 2014; Hempel et al., 2016; Khan et al., 2018; Okamoto et al., 2018; Sekiguchi et al., 2019; Diel et al., 2021; Wakim et al., 2021; Al- Jawahiri et al., 2022; Cho et al., 2022), which have been summarized by Al-Jawahiri et al. (Al- Jawahiri et al., 2022). Although the clinical features of the three Chinese patients were variable, they still fell within the spectrum of *SOX11* syndrome (Table 1). In summary, patients 1 and 2 had highly consistent facial deformities, comprising of prominent forehead, arched eye brow, flat nasal bridge, broad nose, short philtrum, and abnormal ears. In comparison, the facial deformity of patient 3 was much milder. Sparse scalp hair and high hairline were also noticed in both patients 1 and 2. Patients 2 and 3 had DD and ID, and both male patients had short stature and cryptorchidism. In addition, patient 1 harbored the 5^th^ finger clinodactyly and microcephaly was only found in patient 3.

**TABLE 1:** Phenotypic comparison of our patient with reported patients.

	Patient 1	Patient 2	Patient 3	Reported patients (*n* = 58)
General information				
Gender	Male	Female	Male	29 female/28 male
				/1 neonate
Born at full term	+	+	+	12/14
IUGR	+	−	−	5/7
Age	3-years	5.5-years	8-years	—
Birth length (cm)	Unknown	50 (0.2 SD)	49 (−0.8 SD)	—
Birth weight (kg)	2.1 (−3.1 SD)	3.5 (0.7 SD)	3.2 (−0.3 SD)	—
Current height (cm)	79 (−.7 SD)	113 (−0.1 SD)	118 (−2.2 SD)	10 < 2 SD (*n* = 30)
Current weight (kg)	9.6 (−3.3 SD)	18.7 (−0.3 SD)	17 (−2.5 SD)	11 < 2 SD (*n* = 29)
Head circumference (cm)	Normal	Normal	46 (−3.0 SD)	11 < 2 SD (*n* = 28)
Microcephaly	−	−	+	11/28
Short stature	+	−	+	10/30
Facial features				
Prominent forehead	+	+	+	1/7
Arched eye brow	+	+	−	6/16
Flat nasal bridge	+	+	+	3/12
Broad nose	+	+	−	3 with short nose
Short philtrum	+	+	−	7/17
Open mouth	−	+	−	7/15
Abnormal ears	+	+	−	5/7
Micrognathia	+	−	−	2/10
Cleft palate	−	+	−	1/7
Cleft lip	−	+	−	0/15
Neurodevelopment				
DD	−	+	+	36/37
ID	N/R	+	+	38/40
Skeletal malformations				
Clinodactyly	5th finger	−	−	10/55
Hypoplastic nails	−	−	−	6/55
Joint laxity	Fingers	−	−	1/3
Others				
Sparse scalp hair	+	+	−	6/10
High hairline	+	+	+	5/9
Cryptorchidism	+	N/A	+	5/6

DD, developmental delay; ID, intellectual disability; SD, standard deviation; N/A, not applicable; N/R, not reported.

### Identification and *in silico* analysis of the *SOX11* variants in the patients

**FIGURE 2:**
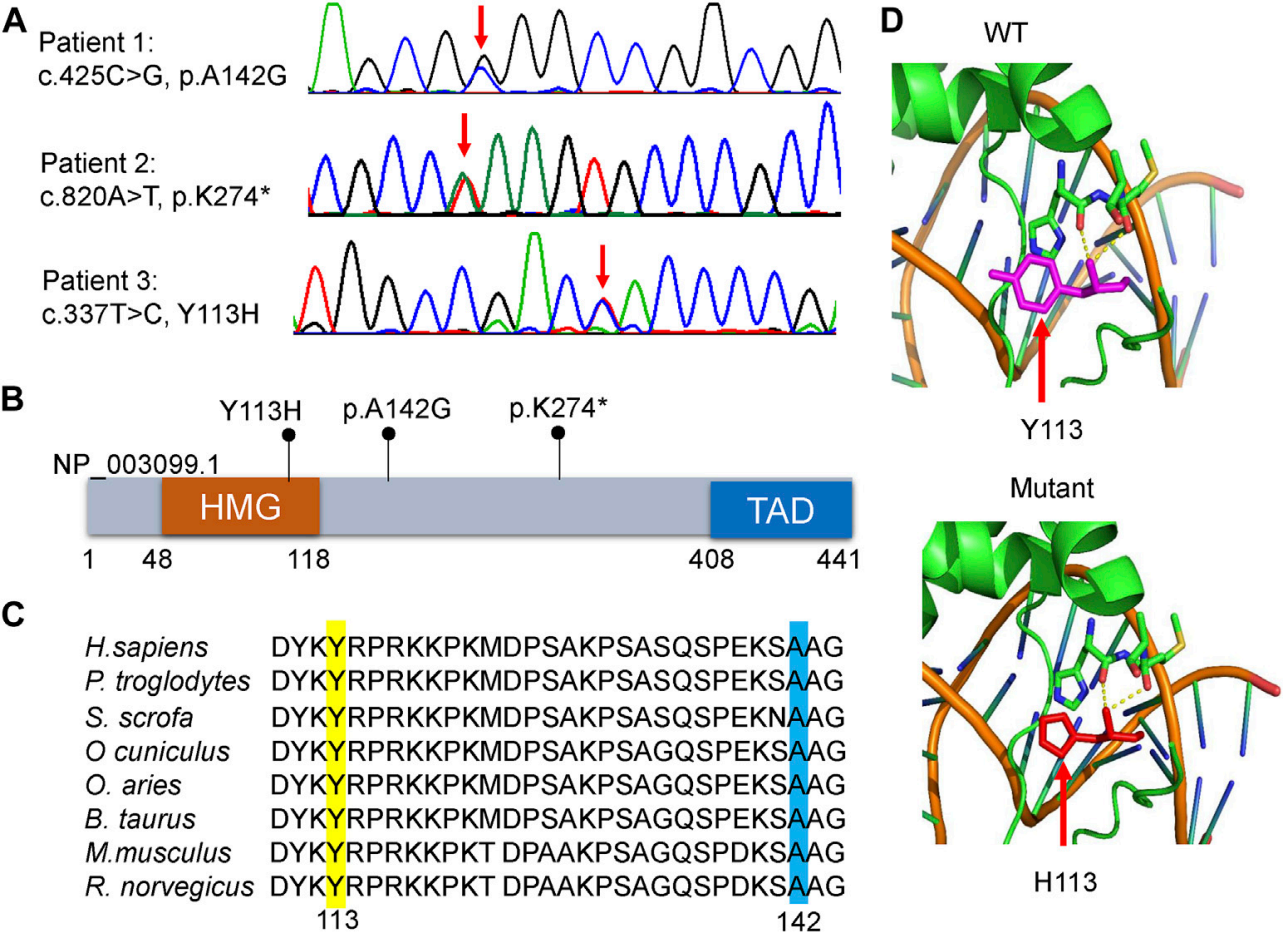
Molecular characteristics of the patients. **(A)** Sanger sequencing revealed that each patient harbored a heterozygous variant in *SOX11* gene (NM_003108.4). Red arrows indicate the variant base. **(B)** Distribution schematic of the three variants of *SOX11* gene identified in this study. Of them, the variant of Y113H localize to the high-mobility group (HMG) domain. TAD, transactivating domain. **(C)** Inter-species amino acid sequence alignment to show the missense variants of Y113H (yellow) and A142G (blue) within a highly conserved region of the protein. **(D)** Solved and predicted three-dimensional models of WT and mutant (Y113H) SOX11. The crystal structure was simulated using the mouse Sox4 HMG domain.

WES identified three heterozygous *SOX11* variants, including the missense variant of c.425C>G, p.A142G in patient 1, the nonsense variant of c.820A>T, p.K274* in patient 2, and the missense variant of c.337T>C, p.Y113H in patient 3. Sanger sequencing confirmed these variants in the patients (Figure 2A), and also revealed the *de novo* status of each variant from the results that their parents were WT in *SOX11* gene. All of the three variants were not reported in previous cases and not included in the known public database (i.e., gnomAD, HGMD and ClinVar), suggesting they were novel.

The K274* is not located in the last exon or within the 3′-most 50 nucleotides of *SOX11*, so it is very likely to lead to nonsense-mediated mRNA decay (Abou Tayoun et al., 2018). The Y113 residue locates at the HMG domain of SOX11 protein (Figure 2B), which is highly conserved in multiple species (Figure 2C). Crystal structure analysis using the mouse Sox4 HMG domain showed the Y113H variant may not alter the secondary structure of SOX11, but is likely to affect its ability to bind DNA (Figure 2D). By contrast, the other two variants of A142G and K274* locate downstream of the HMG domain (Figure 2B), and the A142 residue is also highly conserved (Figure 2C). In addition, the two missense variants of Y113H and A142G are predicted to be deleterious according to various *in silico* tools including PolyPhen-2, MutationTaster2, CADD, and ClinPred (Table 2).

**TABLE 2:** Pathogenicity Predictions for Y113H and A142G variants of *SOX11*.

	c.337T>C, Y113H		c.425C>G, A142G	
*In silico* tools	Score	Prediction	Score	Prediction
PolyPhen-2 (v2.2)	0.999	Probably_damaging	0.904	Possibly_damaging
MutationTaster2	1	Disease_causing	0.976	Disease_causing
CADD (v1.6)	26.1	Damaging	24.4	Damaging
ClinPred	0.993	Pathogenic	0.726	Pathogenic

### Novel missense variants of *SOX11* impair the transcriptional activity

**FIGURE 3:**
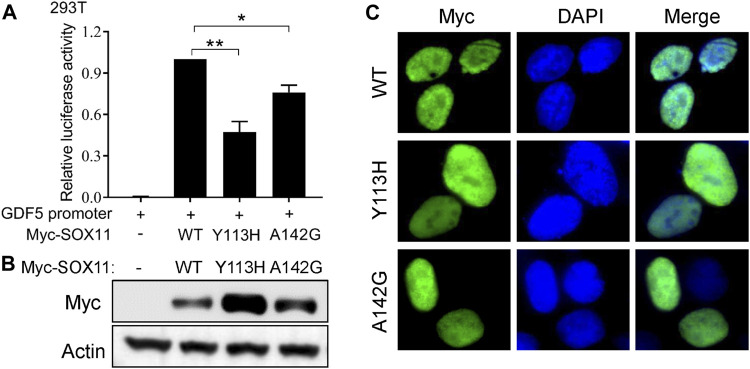
Functional study of the SOX11 missense variants. **(A)** Transcriptional activity of the *GDF5* promoter was determined by luciferase reporter assays in HEK293T cells after co-transfection of the WT or the mutant Myc-*SOX11* and the *GDF5* promoter reporter construct. Data are presented as mean values ±s.e.m. from three independent experiments. *p < 0.05, **p < 0.01, two-tailed Student’s *t*-test. **(B)** The expression level of the WT and the mutant (Y113H and A142G) Myc-SOX11 were evaluated by immunoblotting in HEK293T cells. **(C)** Immunofluorescence analysis to show the subcellular localization of the WT and the mutant (Y113H and A142G) Myc-SOX11.

To further evaluate the impact of the Y113H and A142G variants on SOX11 function, we first performed luciferase assay using a reporter construct containing a fragment of *GDF5* promoter which has been described previously (Tsurusaki et al., 2014). Compared to the with WT *SOX11*, both Y113H and A142G mutants result in decreased transcriptional activities (Figure 3A). *In vitro* experiments in HEK293T cells revealed that both missense variants increased SOX11 expression (Figure 3B), which were also observed in previous studies (Tsurusaki et al., 2014; Hempel et al., 2016). Immunofluorescence analysis showed that the WT and two missense mutant SOX11 protein localized in the nucleus (Figure 3C).

### Identification of the genomic region deletion in patient 1

**FIGURE 4:**
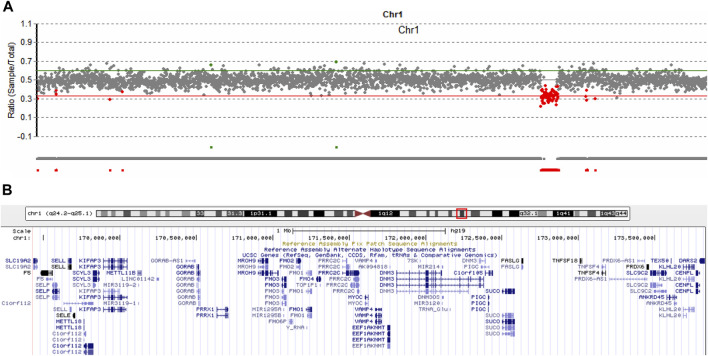
Copy number variation analysis in patient 1. **(A)** Sequencing depth analysis using the WES data showed patient 1 harbored 1q24.2-q25.1 deletion (hg19, chr1:169,433,149-173,827,682). **(B)** Involving genes in the 1q24.2-q25.1 region.

It is estimated that near 5% of patients can have more than one molecular findings, and may cause more serious phenotypic features (Posey et al., 2017). We routinely analyze copy number variation (CNV) of each patient by comparing the read-depth with the WES data from the other samples of the same batch (Yao et al., 2017; Yao et al., 2019). As shown in Figure 4A, patient 1 harbored a 4,300 kb deletion involving the region of 1q24.2-q25.1 (hg19,chr1:169,433,149-173,827,682), which includes multiple disease-causing genes, such as *EEF1AKNMT* (OMIM#617987), *FASLG* (OMIM#134638), *FM O 3* (OMIM#136132), *GORAB* (OMIM#607983), *MYOC* (OMIM#601652), *PIGC* (OMIM#601730), *PRRX1* (OMIM#167420), *SLC19A2* (OMIM#603941), and *TNFSF4* (OMIM# 603,594) (Figure 4B). Deletion of this genomic region has been described in series of patients (Burkardt et al., 2011; Chatron et al., 2015). According to the clinical interpretation of CNV by the American College of Medical Genetics and Genomics (ACMG) and the Clinical Genome Resource (ClinGen) (Riggs et al., 2020), the microdeletion of 1q24.2-q25.1 in this patient was classified as pathogenic. No questionable CNVs were identified in the other two patients.

## Discussion

In this study, we reported three novel *SOX11* variants (Y113H, A142G, and K274*) in Chinese patients. According to the clinical interpretation of genetic variants by the ACMG/AMP 2015 guideline (Richards et al., 2015), The K274* nonsense variant was classified as pathogenic (PVS1+PS2+PM2), *In vitro* experiments show that Y113H and A142G has no effect on the nuclear localization of SOX11 protein, but impairs the transcriptional activity of the target gene *GDF5*. Therefore, the two missense variants are also classified as pathogenic (PS2+PS3+PM2+PP3). Interestingly, rare missense variants in *SOX11* appear to increase its protein expression levels. Such observation is particularly pronounced when the missense variants locate in the HMG domain, including the Y113H variant in our patient and the previously reported variants of K50N, S60P, Y116C and P120H (Tsurusaki et al., 2014; Hempel et al., 2016). It is noticed that variants in the HMG domain more severely impair the transcriptional regulatory activity of *SOX11* than outside the HMG domain (A142G in this study and A176E in ref. 14). These results suggest that the increased protein expression caused by missense variants in HMG domain is a compensatory effect after functional loss. We here provide functional evidence to explain the phenomenon observed by Al-Jawahiri et al. in the gnomAD database that there are far fewer missense variants in the HMG box than outside the HMG domain, including the N-terminal, central, and transactivating domains in SOX11 (Al- Jawahiri et al., 2022).

In addition to the A142G variant in *SOX11*, patient 1 yet harbored microdeletion of 1q24.2-q25.1, which has been described in multiple patients (Burkardt et al., 2011; Chatron et al., 2015). The clinical features resulting from this CNV highly overlap with *SOX11*-related syndrome. For example, Chatron et al. summarized 18 patients with 1q24-q25 deletions and suggested that common clinical phenotypes include IUGR, short stature, microcephaly, delayed bone age, ID, hypertelorism, dysplastic ears, micro- and retrognathia, short hands and feet, brachydactyly, and fifth finger clinodactyly (Chatron et al., 2015). At the moment, it was difficult to determine the contribution of *SOX11* A142 variant and 1q24-q25 deletion to the phenotype of patient 1.

*SOX11* is widely expressed and has an important regulatory role in tissue remodelling during early embryogenesis. Conventional knockout of *Sox11* in mice lead to birth death with severe developmental defects, involving in the malformations in brain, skeleton, eyes, spleen, lung, stomach, pancreas, and heart (Sock et al., 2004; Kato et al., 2015). In particular, *SOX11* is critical for neurogenesis, as it can drive the differentiation of embryonic stem cells into neuronal progenitor cells and the generation of mature neurons and glial cells (Bergsland et al., 2011; Kavyanifar et al., 2018). Such roles of *SOX11* in neurodevelopment have been validated in *Sox11* conditional knockout mice (Wang et al., 2013), *sox11* knockdown xenopus laevis (Hempel et al., 2016), and the *SOX11* ^*+/−*^ heterozygous human embryonic stem cell models (Turan et al., 2019). These molecular mechanism studies can well explain that patients with *SOX11* variants are mainly manifested as DD and/or ID. Consistently, patients 2 and 3 in this study had both DD and ID. However, patients with neither DD nor ID were also reported in previous studies (i.e., case 9 who harbored H109P missense variant in the HMG domain in ref. 14), which may also be true in patient 1 of this study. These indicate a high degree of heterogeneity in clinical features caused by *SOX11* variants.

Typical CSS, which is also referred to as BAFopathy, is caused by variants in the subunit of BAF complex, including *ARID1A* (OMIM#603024), *ARID1B* (OMIM#614556), *SMARCA4* (OMIM#603254), *SMARCB1* (OMIM#601607), *ARID2* (OMIM#609539), and *SMARCE1* (OMIM#603111) (Bogershausen and Wollnik, 2018). Due to the clinical phenotypic similarity, *SOX11* was considered as the new causative gene of CSS (Tsurusaki et al., 2014; Hempel et al., 2016). However, with the number of patients increased, the differences between patients with *SOX11* variation and CSS gradually became apparent. Al-Jawahiri et al. revealed that *SOX11* syndrome has distinct clinical features from *ARID1B*-related CSS. Firstly, *SOX11* mutant patients with microcephaly tend to be associated with oculomotor apraxia or abnormal eye morphology, while the *ARID1B*-related CSS patients usually have a coarse face. Secondly, deformity of the fifth fingers is an identifiable feature of CSS but do not appear to be specific to *SOX11* syndrome. Thirdly, nearly one in five of the *SOX11* mutant patients had hypogonadotrophic hypogonadism which was rare reported in CSS patients. Additionally, they uncovered a distinctive pattern of blood DNA methylation in the patients with *SOX11* variants, and thus generated a *SOX11* variation-related episignature model which was also failed to identify the BAFopathy complex samples. These results strongly suggest *SOX11* syndrome and CSS were two groups of clinically and molecularly distinct diseases (Al- Jawahiri et al., 2022). In our study, none of the patients had coarse facies and hypoplastic nails, which are the core features of CSS. Moreover, the phenotypic summary of reported patients showed only about one-third of patients had microcephaly, and one-tenth had nail dysplasia (Table 1), also suggesting differences between *SOX11*-related phenotypes and classical CSS.

In conclusion, our study identified three novel *SOX11* variants and elucidated the damage effect of two missense variants on SOX11 protein *via in vitro* experiments. This is the first report of Chinese patients with *SOX11*-related CSS9. Our study provides new evidence to support the observation that the clinical phenotype caused by *SOX11* variants is highly heterogeneous and differs from classical CSS to some extent.

## Data Availability

The datasets for this article are not publicly available due to concerns regarding participant/patient anonymity. Requests to access the datasets should be directed to the corresponding author.
